# Optimized protocol for MALDI MSI of N-glycans using an on-tissue digestion in fresh frozen tissue sections

**DOI:** 10.1038/s41598-023-29560-6

**Published:** 2023-02-16

**Authors:** Andrej Grgic, Kasper K. Krestensen, Ron M. A. Heeren

**Affiliations:** The Maastricht MultiModal Molecular Imaging (M4I) Institute, Division of Imaging Mass Spectrometry (IMS), Maastricht University, 6229 ER Maastricht, The Netherlands

**Keywords:** Molecular imaging, Mass spectrometry, Glycobiology

## Abstract

Glycans play an important role in biology with multiple cellular functions ranging from cell signaling, mobility and growth to protein folding and localization. The N-glycosylation state within a tissue has been found to vary greatly between healthy and diseased patients and has proven to have an important clinical diagnostic value. Matrix assisted laser-desorption ionization (MALDI) mass spectrometry imaging (MSI) allows for untargeted analysis of biomolecules, including N-glycans, on a tissue section and provides a spatial context of the analyte. Until now, N-glycans have been predominantly analyzed using MALDI MSI on formalin-fixed paraffin embedded (FFPE) tissue sections, however this greatly reduces the clinical applicability, as the FFPE embedding process alters the biological environment of the tissue. Here we developed a protocol that allows for MALDI MSI of N-glycans from fresh frozen tissue that matches the current standard of FFPE analysis. By optimizing several steps in the sample preparation, we see orders of magnitude increase in signal intensity. Furthermore, this method limits delocalization of released N-glycans, thus improving the effective spatial resolution of the label-free molecular images. This protocol provides a novel perspective towards clinical application of MALDI MSI and capitalizes on the diagnostic value of N-glycan analysis.

## Introduction

Glycobiology describes the role and structure of carbohydrates, or glycans, in biology. In a cellular context, glycans are found as long linear disaccharide-repeats or as glycoconjugates, where the glycans are covalently linked to lipids or proteins, called glycolipids and glycoproteins respectively^1^. Glycoproteins have been found to play numerous important roles in the cell including, intracellular structure, protein folding and localization, association with cell surface receptors, affecting cell mobility and cell growth, as well as cell–cell signaling and immune cell functions^2^. However, as protein glycosylation is regulated both genetically and epigenetically, the glycosylation status of the cell is highly variable and has found to be an important factor to consider in both health and disease^1,3^.

Glycosylation is one of the most common forms of post-translational modifications and results in either N-linked glycans or O-linked glycans depending on the modified amino acid. For the scope of the method described here, only N-linked glycans are considered. The process of N-linked glycosylation happens during translation where the donor glycan is attached to an asparagine (N) residue on the growing polypeptide chain in the endoplasmic reticulum^4^. This attachment always happens at the consensus sequence N-X-S/T, where X is any amino acid other than proline^5^. The attached N-glycan structure is finalized by several enzymatic modifications as the protein travels through the Golgi apparatus. Peptide N-glycosidase (PNGase F) is one of the enzymes that regulate the glycosylation status in the cell through cleavage of the innermost glycosidic bond between the modified asparagine and the attached glycan^6^. This results in the N-glycan being released and allows for analysis of the N-glycosylation status for research purposes.

Matrix assisted laser-desorption ionization (MALDI) mass spectrometry imaging (MSI) is a powerful analytical tool that allows for untargeted analysis and localization of biomolecules within a tissue section^7^. A big advantage of MALDI MSI is that different molecular species can be analyzed on the same instrument by simply changing the sample preparation. Thus, information about the proteome, lipidome and metabolome can be obtained and put into the histological context of the same tissue section with the help of complementary staining methods^8^. MALDI MSI has also been extended to N-glycan imaging of both healthy and diseased tissue and specific N-glycan patterns have shown to be important markers in several cancer types, as well as other diseases^9–13^. To best utilize the tissue banks available at most research institutions, many of the workflows for MALDI MSI have been optimized for formalin-fixed paraffin embedded (FFPE) tissue sections. However, there are several drawbacks to using FFPE samples including, non-standardized embedding procedures making direct comparisons between same tissue-types from different institutions difficult. Furthermore, the embedding process depletes the tissue of many biomolecules and crosslinks and denatures all proteins in the tissue, potentially altering the biological environment compared to fresh frozen tissues. On the other hand, fresh frozen tissues are preferred for clinical applications as the freezing process is rapid which allows for analysis mid-surgery, such as assessing tumor margin of removed tissue. Additionally, the biological environment is kept mostly intact as proteins and other biomolecules are frozen in their native state^14^. While workflows for imaging N-glycans on fresh frozen tissue have been reported, the obtained spectra had significantly worse intensity and less identified peaks compared to the FFPE counterpart^5^.

Based on the current state of N-glycan imaging, we saw an unmet need for a workflow to image N-glycans with MALDI-MSI in fresh frozen tissue to pave the road for potential clinical implementation of this analysis. Here we present a protocol for fresh frozen N-glycan imaging of which the results match the current gold standard of FFPE-tissue imaging.

## Materials and methods

### Chemicals

Water (HPLC and ULC/MS grade), ethanol, acetonitrile (ACN), trifluoroacetic acid (TFA), and xylene were obtained from Biosolve BV (Valkenswaard, The Netherlands). $\mathrm{\alpha }$-Cyano-4-hydroxycinnamic acid (CHCA), citraconic anhydride, and potassium sulfate were obtained from Sigma Aldrich (St. Louis, MI, USA). Hydrochloric acid fuming 37% was obtained from Supelco (Bellefonte, PA, USA). Basic N-glycan imaging kit was obtained from GlycoPath Inc. (Charleston, SC, USA).

### Samples

Patient samples (n = 2) were procured in the framework of prospective local biobank programs that were initiated in 2017 (Maastricht) and 2009 (Aachen). For MSI, the workflow for cryopreservation of tissues was optimized to minimize time between surgical excision and diagnostic processing of specimens by the pathologist. The biobank programs were approved by local ethical committees (Maastricht #16-4-153, Aachen EK 206/09), and all patients scheduled for surgical treatment of hepatobiliary tumors were eligible for participation. Participating patients provided written informed consent for storage and research use of their specimens.

The study on the TMA (n = 1) sample was approved by the institutional ethics review board (Ethics Committee of the Technical University of Munich Faculty of Medicine, Protocol Number 403/17S). All experiments using human patient samples were conducted in compliance with the respective institutional guidelines.

Fresh frozen mouse kidney (n = 1), spleen (n = 2) and brain (n = 1) samples were obtained from Johns Hopkins University School of Medicine. All animal experiments were performed with appropriate ethical approval (2014-108 at GROW Maastricht University and A3272-01 at the Johns Hopkins University) and in compliance with the respective institutional guidelines.

### Sample preparation

FFPE tissue microarray (TMA) of cholangiocarcinoma (CCA) and pancreatic ductal adenocarcinoma (PDAC) were sectioned at 5 µm thickness and mounted on Intellislides (Bruker Daltonics GmbH, Bremen, Germany). Fresh frozen tissues were sectioned at 12 µm thickness and mounted on indium tin oxide (ITO) coated glass slides (Delta Technologies Ltd, Loveland, CO, USA). All fresh frozen samples were measured in duplicate or more.

For FFPE tissue, the protocol provided with the GlycoPath Basic N-Glycan Imaging Kits (GlycoPath Inc., Charleston, SC, USA) was used throughout^5^. Briefly, the TMA sections were dewaxed in the oven by heating at 60 °C for 60 min. Following dewaxing, the washing steps consisted of 2 times for 3 min in xylene, 2 times for 1 min in 100% ethanol, 1 time for 1 min in 95% ethanol, 1 time for 1 min in 70% ethanol, and 2 times in distilled water for 3 min. Afterwards, antigen retrieval was performed in a citraconic anhydride buffer at pH = 3. Slides were then cooled and dried in a desiccator, which was followed by a PNGase F application with HTX M3+ sprayer (HTX Technologies LLC, Carrboro, USA). Slides were then incubated for 2 h at 37.5 °C. Finally, the CHCA matrix was applied according to GlycoPath protocol with the HTX M3+ sprayer^5^.

For fresh frozen tissue the developed protocol starts with the washing and fixation steps. The slide with tissue sections were immersed 2 times for 2 min in ice-cold 100% ethanol, 1 time for 1 min in ice-cold 96% ethanol, 1 time for 1 min in ice-cold 70% ethanol, and 2 times for 2 min in ice-cold HPLC grade water. The slides were subsequently dried in a desiccator and teaching points were applied with a white paint marker. Slides were scanned prior to antigen retrieval. Antigen retrieval was performed using the Retriever 2100 (Aptum Biologics Ltd, Rownhams, UK) for 20 min at 121 °C. The citraconic anhydride buffer (pH = 3) was prepared as described by Drake et al.^5^ and in the GlycoPath protocol. The slide holder was taken out of the antigen retriever and cooled in an ice bath for 5 min. Half of the buffer was then replaced with HPLC grade water and slide holder was placed back to cool further in an ice bath. This was repeated two more times after which the slides were rinsed with HPLC grade water and dried in a desiccator. PNGase F was freshly prepared prior to the enzyme application with HTX M3+ sprayer using the following settings: temperature = 45 °C, nozzle velocity = 1200 mm/min, flow rate = 30 µL/min, PNGase F concentration = 0.1 µg/µL, number of passes = 8, track spacing = 2.5 mm, and nitrogen gas pressure of 10 psi. The incubation chamber was prepared by placing paper wipes soaked with saturated potassium sulfate solution on the bottom of a glass petri dish with two additional wipes being rolled into a support-structure that kept the slides away from the soaked bottom of the incubation chamber. Furthermore, 5 g of solid potassium sulfate was added on the top of wipes inside the incubation chamber. The incubation chamber was preheated in oven to the 37.5 °C for 30 min prior to the placement of the slides in the incubation chamber. Slides were transferred into an incubation chamber as soon as enzyme application was done, and they were left to incubate overnight (16 h). Slides were then placed in a desiccator to dry prior to the matrix application. CHCA matrix was applied with the HTX M3+ sprayer (HTX Technologies LLC, Carrboro, USA) using the following settings: temperature = 75 °C, nozzle velocity = 1200 mm/min, flow rate = 120 µL/min, CHCA concentration = 10 µg/µL, number of passes = 4, track spacing = 1.5 mm, and nitrogen gas pressure of 10 psi.

### MALDI MSI experiments

MALDI-MSI data of the FFPE tissue dataset were acquired on a timsTOF fleX in positive polarity (Bruker Daltonik GmbH, Germany) at a pixel size of 20 × 20 µm, using 300 shots and a laser frequency of 10 kHz. MALDI-MSI data of the fresh frozen tissue dataset were also acquired at the same polarity, number of laser shots, and a laser frequency, but with a pixel size of 16 × 16 µm. The instrument is equipped with a Nd:YAG laser emitting at 355 nm with a laser focus diameter of 5 µm. Mass calibration was performed using red phosphorus before the imaging experiments.

### Data analysis

Majority of the data analysis was performed with SCiLS lab 2022a (SCiLS GmbH, Bremen, Germany). Generated images were all TIC normalized and exported from SCiLS lab. Average spectra from both, the FFPE and the FF, MALDI MSI experiments were exported from SCiLS lab and imported in mMass where peak picking was performed^15^. Settings used for peak picking in mMass were as followed: S/N threshold of 5, relative intensity threshold of 0.5%, picking height was set at 75, and baseline and deisotoping functions were applied.

### Glycan identification

All identifications were made tentatively based on an accurate mass and data from GlycoPath database generated with MALDI FT-ICR measurements. Access to the database is provided with purchase of the basic N-glycan imaging kit.

### Ethics approval

Local ethical committees approved the biobank programs (Maastricht #16-4-153, Aachen EK 206/09), and informed consent was obtained from all individual participants whose materials were analyzed in the study.

### Informed consent statement

Patient consent was waived due to the use of archival data of anonymous nature that does not disclose patients’ identities. The patient consent can be waived, as it is contemplated by the Bavarian state law (available only in German), in the Bavarian Hospital Act (BayKrG) section, published on 28 March 2007 (https://www.gesetze-bayern.de/Content/Document/BayKrG/true, accessed on 28 October 2021), on the article 27 of Data protection, Section 4.

### Institutional review board statement

The study was approved by the institutional ethics review board (Ethics Committee of the Technical University of Munich Faculty of Medicine, Protocol Number 403/17S).

## Results and discussion

### Optimized MALDI MSI sample preparation and analysis workflow

**Figure 1:**
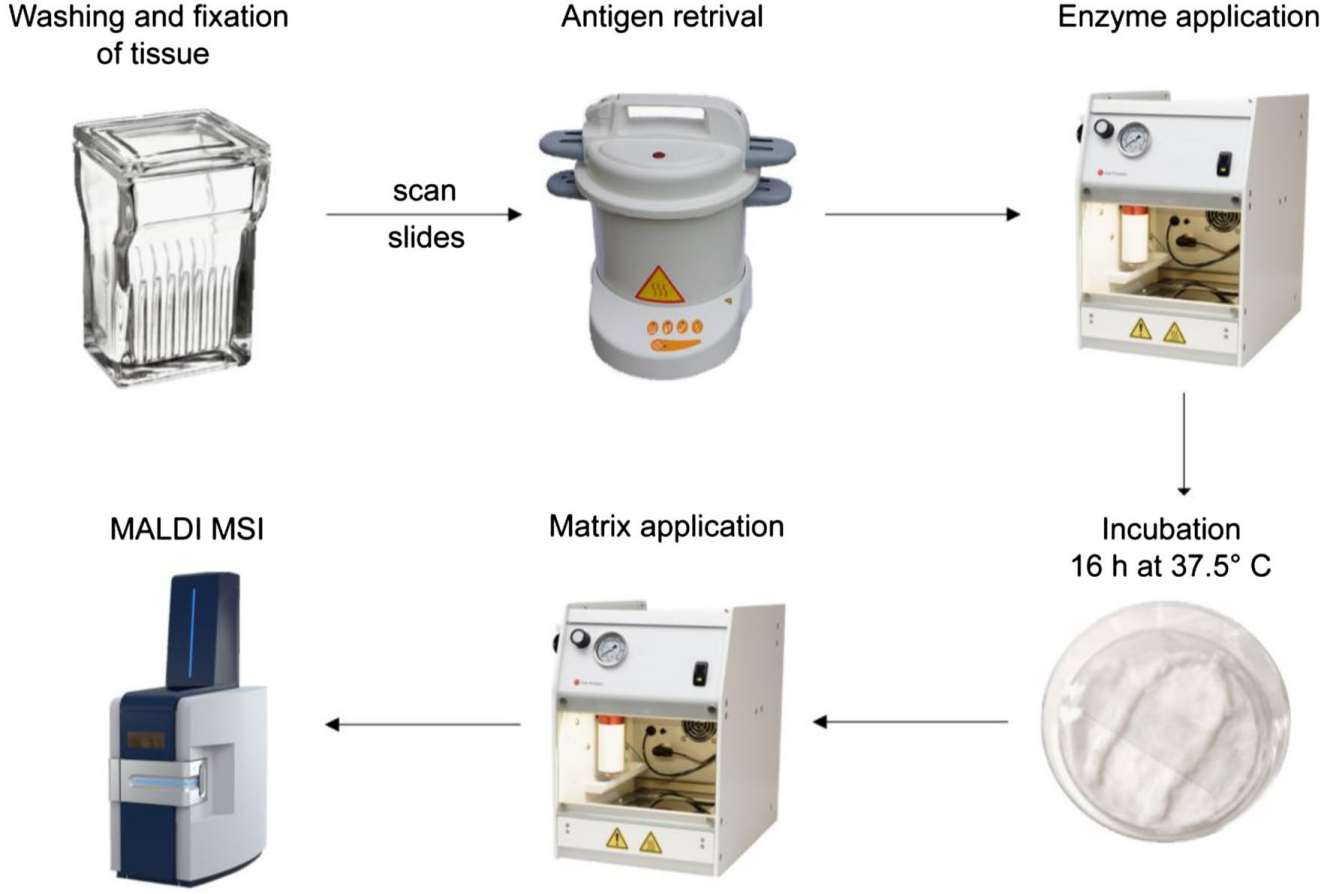
Experimental overview of N-glycan imaging protocol from sample preparation to MALDI MSI.

For the optimized fresh frozen N-glycan MALDI MSI protocol, several steps were optimized to achieve the best possible results. An overview of the workflow can be seen in Fig. 1.

First, the tissue washing was optimized by reducing the number of washes and introducing ice-cold solvents as based on a recently published optimized MALDI MSI protocol for trypsin digestion. It was reported that using ice-cold solvents for washing reduced delocalization and reducing the number of washes reduced the sample preparation time^16^.

Second, an antigen retrieval step was added prior to enzyme application (Fig. 1). Antigen retrieval is a necessary step when handling FFPE tissue sections, as proteins in the tissue are highly cross-linked by the formalin fixation and any potential epitopes are therefore unavailable for enzymatic digest or antibody staining. By heating the tissue above 95 °C in an appropriate buffer, the formalin cross-links are broken, and antigenicity of the proteins are reconstituted^17^. As a buffer, citrate at pH = 6 is most commonly used, but studies have shown that using a citraconic anhydride buffer at pH = 3 instead can result in an improved antigen retrieval^18^. For N-glycan MALDI MSI on FFPE tissues specifically, citraconic anhydride buffer is preferred and is therefore used in this protocol. Antigen retrieval is usually not a necessary step when analyzing fresh frozen tissue as the proteins are frozen in their native conformation. However, recently it was shown that briefly heating the tissue to 95 $^\circ$C to denature the proteins, and thereby expose more cleavage sites, resulted in a more efficient digestion using trypsin^16^. This same principle was applied here to optimize fresh frozen tissue analysis and a full antigen retrieval step is included instead of the short heating step reported previously.

Third, another point of optimization was the addition of dissolved potassium sulfate to the humidity chamber during the overnight digestion with PNGase F. Using a saturated potassium sulfate solution helps maintain a higher relative humidity of about 96% in the chamber, thereby reducing potential spatial delocalization of the released N-glycans after cleavage^19^.

Fourth, the incubation time with PNGase F was increased from two to 16 h. Based on in-house optimization for digestion with trypsin, two hours of incubation was insufficient for proper digestion of the sample with trypsin resulting in low sensitivity. Therefore, the incubation time was increased in an effort to increase the amount of released N-glycans and thereby increase sensitivity.

Finally, the spray method for the MALDI matrix was altered to be less wet and thereby reduce possible delocalization. This was done by reducing the number of passes from 10 to four, while adjusting the remaining parameters to fit as mentioned in the methods.

### MALDI MSI of fresh frozen tissue

**Figure 2:**
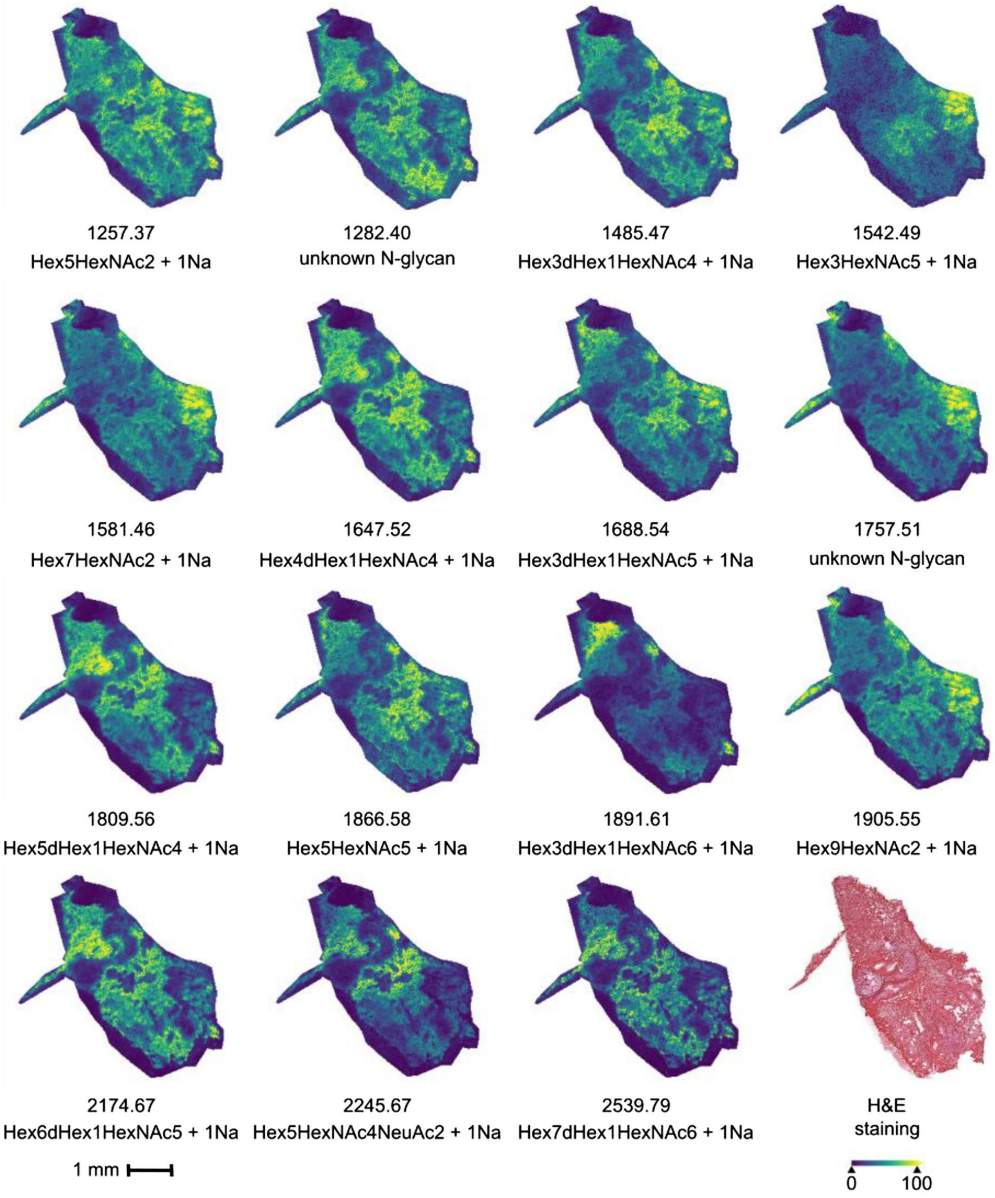
MALDI MSI images of selected potential N-glycans in fresh frozen CCA tissue. The spatial distribution of 15 potential N-glycan *m/z*-values detected in the same tissue section is highlighted. Tentative identification were provided where possible. Measurement was done in positive ion-mode and with a pixel size of 16 × 16 µm.

Fresh frozen cholangiocarcinoma tissue was treated with the optimized N-glycan imaging protocol and compared with FFPE PDAC/CCA TMA tissue treated with the standard FFPE N-glycan imaging protocol provided from GlycoPath to test the new sample preparation and MALDI MSI imaging protocol. The samples were imaged on a timsTOF fleX in positive ion-mode to obtain the molecular image of released N-glycans. For both samples of human CCA tissue, all replicates of digestion and MALDI-MSI measurement showed similar results, results shown are from one tissue section only (Fig. 2).

Figure 2 shows the spatial distributions of 15 detected potential N-glycans across the measured mass range of *m/z* 900–3000 acquired with a pixel size of 16 × 16 µm. Comparisons of mass spectra comparing the old fresh frozen protocol with the new optimized protocol can be seen in supplementary Fig. S1. MALDI images from the old protocol showed no distinguishable N-glycan features (not shown) and comparing the absolute intensity spectra clearly show an increased intensity of several orders of magnitude, when comparing the old protocol with the optimized fresh frozen protocol (Supplementary Fig. S1). Interestingly, each of the highlighted N-glycan peaks in Fig. 2 show a unique spatial distribution in the tissue. While some masses are more ubiquitously present (*m/z* 1257.37, 1485.47, 1647.52) other masses are highly confined to a specific area in the tissue (*m/*z 1891.61, 1912.50). This differential spatial distribution between specific masses suggests a different cellular function and reiterates the need for high-resolution, high-sensitivity imaging of N-glycans in fresh frozen tissue. The wide applicability and robustness of the new protocol was supported by measurements of different tissue types, including fresh frozen mouse kidney, spleen and brain samples. Spectra from the different tissues, including a separate sample of human CCA can be seen in supplementary Fig. S2.

Some of the optimizations made to the protocol were to limit delocalization of released N-glycans. This decrease is evident when comparing the images obtained from the optimized fresh frozen MALDI MSI protocol to images obtained from comparable FFPE PDAC/CCA TMA tissue prepared with the standard GlycoPath FFPE protocol (Supplementary Fig. S3). Figure 2 shows a clear distribution of potential N-glycans within the tissue boundaries with almost no detectable signal of delocalization outside or at the border of the tissue. In comparison, the FFPE TMA images show considerable signal outside of the TMA core for several *m/z*-values corresponding with potential N-glycans, indicating that some delocalization of the released N-glycans is happening.

**Figure 3:**
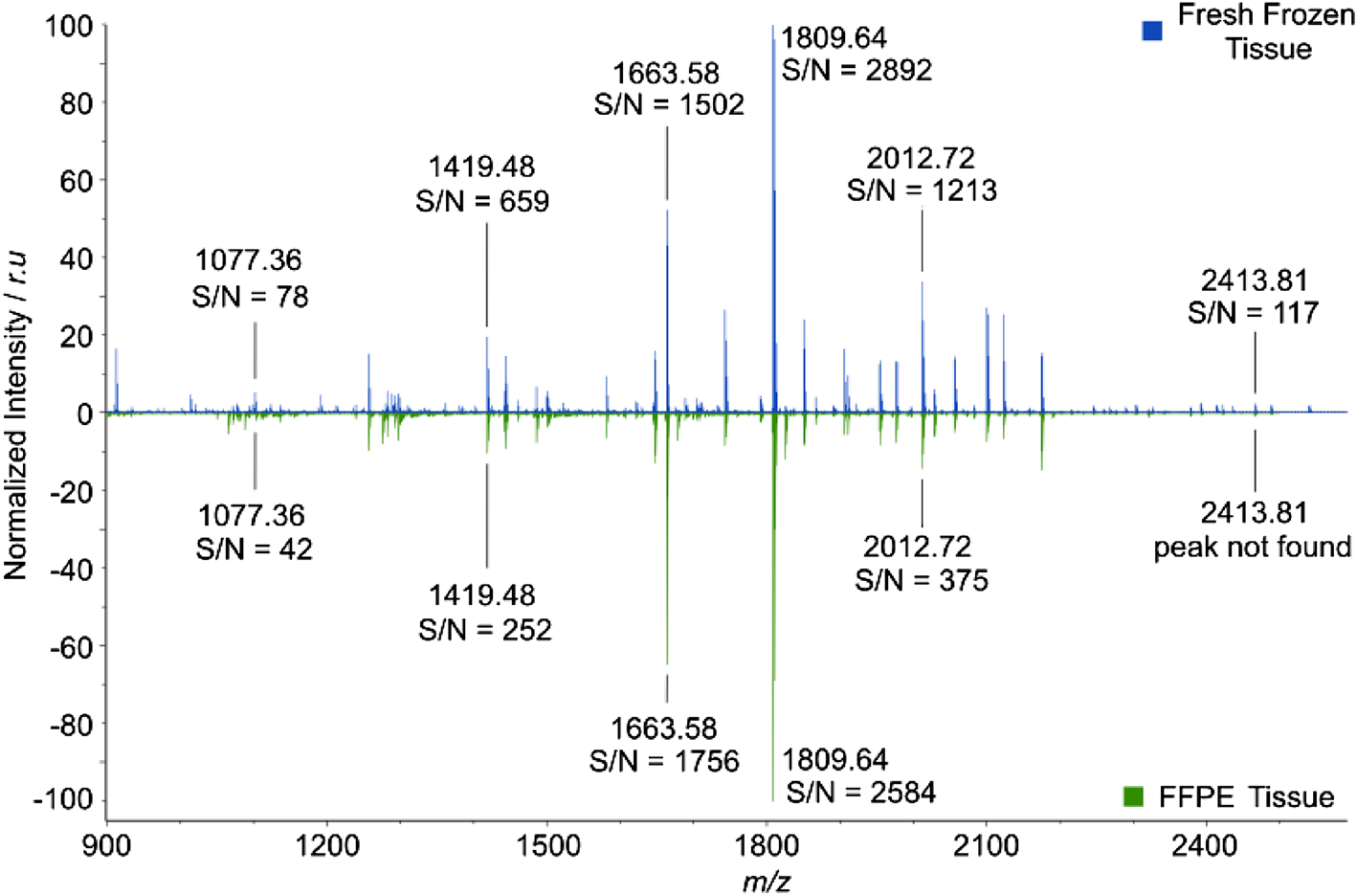
Comparison of normalized average mass spectra acquired from fresh frozen CCA tissue sections (top, blue) and FFPE tissue TMA of PDAC/CCA (bottom, green). Six detected *m/z*-values matching potential N-glycans are annotated with corresponding signal-to-noise ratio (S/N) values for fresh frozen and FFPE measurement. Peak at *m/z* 2413.81 was not detected in FFPE tissue.

To further compare the optimized fresh frozen N-glycan imaging protocol to the gold standard of FFPE tissue, the resulting spectrum from a fresh frozen MALDI MSI experiment was compared with a spectrum from an FFPE MALDI MSI experiment. The resulting MALDI MSI spectra are shown in Fig. 3.

Figure 3 shows a comparison of normalized N-glycan imaging spectra from fresh frozen CCA tissue and FFPE tissue. Following peak picking, 211 peaks were observed in the fresh frozen tissue compared with 215 peaks in the FFPE tissue. Focusing on N-glycans across the whole *m/z*-range, 103 potential N-glycan peaks were detected in the fresh frozen tissue, with 32 of those being unique to the fresh frozen spectrum, compared to 96 potential N-glycans in the FFPE tissue, with 23 unique peaks. As can be seen in the figure, the observed peaks between two spectra are quite comparable and in-fact, 73 of the 128 detected potential N-glycans peaks are overlapping between the spectra. An overview of detected potential N-glycans can be seen in supplementary Fig. S4 and supplementary table S1. Signal-to-noise ratio (S/N) of selected *m/z*-values corresponding with potential N-glycan masses are highlighted in the figure with the S/N being higher in the fresh frozen spectrum for five out of six *m/z*-values.

Focusing on the edges of the *m/z-*range (Supplementary Fig. S5), it is apparent that the FFPE spectrum contains more peaks in the lower mass range from *m/z* 900–1200, however these are mostly matrix peaks as can be seen in Supplementary Fig. S5a, where N-glycan peaks in the region have been highlighted. All five potential N-glycan peaks detected are overlapping between the fresh frozen and FFPE spectra. On the other hand, in the higher mass range from *m/z* 1900–2500, fresh frozen tissue clearly detected more peaks (21 peaks vs 11 peaks in FFPE spectrum) and based on the *m/z*-values detected, these are likely to be unidentified N-glycans compared to matrix peaks (Supplementary Fig. S5b). CHCA peaks in this *m/z-*range are expected to have a mass with decimal point around 0.0, 0.1 or 0.2, while N-glycan peaks are expected to be around 0.7 moving to 0.8 as the *m/z* increases. Thus, the optimized protocol for MALDI MSI of N-glycans delivers higher sensitivity both in terms of signal and number of N-glycan corresponding peaks.

## Conclusions

Here, we have presented a novel protocol for MALDI MSI of N-glycans in fresh frozen tissue. We have shown that introduction of an antigen retrieval step into the sample preparation workflow, as well as optimization of the conditions under which the tissue is digested, is highly beneficial and improves signal intensity as well as number of detected N-glycans. A visual reduction in delocalization of the released N-glycans is also observed, even compared to the current FFPE tissue protocol. The optimized fresh frozen tissue on-slide digestion protocol yields a result comparable to the current gold standard of FFPE on-slide digestion protocols for N-glycans. Furthermore, the higher number of N-glycan related peaks detected in the mass spectrum also show an increase in S/N ratios. This effect was shown to be more pronounced as we move toward the higher end of the *m/z*-range.

## Supplementary Information

Supplementary Information.

## Data Availability

Data supporting the conclusions presented in the paper can be supplied by corresponding author upon request.
