# Supplementary material for: Optimized protocol for MALDI MSI of N-glycans using an on-tissue digestion in fresh frozen tissue sections
Source: Sci Rep. 2023 Feb 16;13:2776. doi: 10.1038/s41598-023-29560-6 (PMC9935634; doi:10.1038/s41598-023-29560-6)
Supplement: Supplementary file 1 — Supplementary Information. [file 41598_2023_29560_MOESM1_ESM.docx]

**Supplementary information:**

**Optimized protocol for MALDI MSI of N-Glycans using an on-tissue digestion in fresh frozen tissue sections**

Andrej Grgic^1#^, Kasper K. Krestensen^1#^, Ron M.A. Heeren^1*^

^1^The Maastricht MultiModal Molecular Imaging (M4I) institute, Division of Imaging Mass Spectrometry (IMS), Maastricht University, 6229 ER Maastricht, The Netherlands

^#^ These authors have contributed equally

* Corresponding author:

Prof. Ron M.A. Heeren

Email: [r.heeren@maastrichtuniversity.nl](mailto:r.heeren@maastrichtuniversity.nl)

**Table of contents**

**Table S1**: All observed N-glycans peaks with corresponding tentative identifications where possible. 2

**Figure S1**: Comparison of MALDI MSI spectra from N-glycan imaging comparing the old protocol with the new optimized protocol for fresh frozen imaging 5

**Figure S2**: MALDI MSI spectra from N-glycan imaging with the optimized protocol on four different tissue types 6

**Figure S3:** Comparison of delocalization between MALDI MSI measurements of N-glycans on fresh frozen and FFPE tissue sections. 7

**Figure S4**: Overview of potential N-glycans detected across the m/z-range 8

**Figure S5**: Low and high m/z-range of normalized average mass spectra acquired from fresh frozen CCA tissue sections and FFPE tissue TMA of PDAC/CCA 9

# **Table S1**: All observed N-glycans peaks with corresponding tentative identifications where possible.

| *m/z* | Tentative identification | FF Tissue | FFPE Tissue | Theoretical *m/z* | Mass Accuracy / ppm |
| --- | --- | --- | --- | --- | --- |
| 915.31 |  | X | X |  |  |
| 917.32 |  |  | X |  |  |
| 933.32 |  | X | X |  |  |
| 1077.36 |  | X | X |  |  |
| 1079.37 | Hex3dHex1HexNAc2 + 1Na | X | X | 1079.3749 | -4.540 |
| 1095.37 | Hex4HexNAc2 + 1Na | X | X | 1095.3698 | 0.183 |
| 1118.38 |  | X | X |  |  |
| 1136.40 | Hex3HexNAc3 + 1Na | X | X | 1136.3964 | 3.168 |
| 1214.67 |  | X |  |  |  |
| 1236.65 |  | X |  |  |  |
| 1239.41 |  | X | X |  |  |
| 1257.42 | Hex5HexNAc2 + 1Na | X | X | 1257.4226 | -2.068 |
| 1264.44 |  |  | X |  |  |
| 1273.40 |  |  | X |  |  |
| 1280.44 |  | X | X |  |  |
| 1282.45 |  | X | X |  |  |
| 1298.45 | Hex4HexNAc3 + 1Na | X | X | 1298.4492 | 0.616 |
| 1310.45 |  |  | X |  |  |
| 1339.47 | Hex3HexNAc4 + 1Na | X | X | 1339.4757 | -4.255 |
| 1419.48 | Hex6HexNAc2 + 1Na | X | X | 1419.4755 | 3.170 |
| 1426.49 |  | X | X |  |  |
| 1435.45 |  | X | X |  |  |
| 1442.49 |  | X | X |  |  |
| 1444.51 | Hex4dHex1HexNAc3 + 1Na | X | X | 1444.5071 | 2.008 |
| 1460.50 | Hex5HexNAc3 + 1Na | X | X | 1460.502 | -1.369 |
| 1472.50 |  | X |  |  |  |
| 1483.52 |  | X | X |  |  |
| 1485.53 | Hex3dHex1HexNAc4 + 1Na | X | X | 1485.5336 | -2.423 |
| 1501.53 | Hex4HexNAc4 + 1Na | X | X | 1501.5286 | 0.932 |
| 1518.48 |  |  | X |  |  |
| 1524.49 |  | X | X |  |  |
| 1529.52 |  | X | X |  |  |
| 1540.46 |  |  | X |  |  |
| 1542.55 | Hex3HexNAc5 + 1Na | X |  | 1542.5551 | -3.306 |
| 1545.56 |  | X |  |  |  |
| 1562.53 |  | X | X |  |  |
| 1573.55 |  | X |  |  |  |
| 1581.53 | Hex7HexNAc2 + 1Na | X | X | 1581.5283 | 1.075 |
| 1584.54 |  | X |  |  |  |
| 1597.50 |  |  | X |  |  |
| 1604.54 |  | X | X |  |  |
| 1606.56 | Hex5dHex1HexNAc3 + 1 Na | X | X | 1606.5599 | 0.062 |
| 1622.55 | Hex6HexNAc3 + 1Na | X | X | 1622.5548 | -2.958 |
| 1629.57 |  | X | X |  |  |
| 1631.59 |  |  | X |  |  |
| 1645.57 |  | X | X |  |  |
| 1647.58 | Hex4dHex1HexNAc4 + 1Na | X | X | 1647.5865 | -3.945 |
| 1661.56 |  |  | X |  |  |
| 1663.58 | Hex5HexNAc4 + 1Na | X | X | 1663.5814 | -0.842 |
| 1675.58 |  | X | X |  |  |
| 1679.55 |  | X | X |  |  |
| 1688.61 | Hex3dHex1HexNAc5 + 1Na | X | X | 1688.613 | -1.777 |
| 1691.60 |  | X | X |  |  |
| 1704.60 | Hex4HexNAc5 + 1Na | X | X | 1704.608 | -4.693 |
| 1708.59 |  | X | X |  |  |
| 1733.59 |  | X |  |  |  |
| 1735.60 |  | X | X |  |  |
| 1743.58 | Hex8HexNAc2 + 1Na | X | X | 1743.5811 | -0.631 |
| 1757.58 |  | X |  |  |  |
| 1759.56 |  | X | X |  |  |
| 1775.60 |  | X | X |  |  |
| 1791.63 |  | X | X |  |  |
| 1807.62 |  | X | X |  |  |
| 1809.64 | Hex5dHex1HexNAc4 + 1Na | X | X | 1809.6393 | 0.387 |
| 1817.62 |  | X |  |  |  |
| 1823.60 |  |  | X |  |  |
| 1825.62 |  | X | X |  |  |
| 1831.56 |  | X | X |  |  |
| 1835.65 |  | X |  |  |  |
| 1837.63 |  | X | X |  |  |
| 1848.65 |  |  | X |  |  |
| 1850.65 | Hex4dHex1HexNAc5 + 1Na | X | X | 1850.6393 | 5.782 |
| 1866.65 | Hex5HexNAc5 + 1Na | X | X | 1866.6608 | -5.786 |
| 1891.69 | Hex3dHex1HexNAc6 + 1Na | X | X | 1891.6924 | -1.269 |
| 1894.69 |  | X |  |  |  |
| 1905.63 | Hex9HexNAc2 + 1Na | X | X | 1905.6339 | -2.047 |
| 1910.69 |  | X | X |  |  |
| 1911.58 | Hex5dHex1HexNAc4 + 1SO4 + 2Na | X | X | 1911.5781 | 0.994 |
| 1921.65 |  |  | X |  |  |
| 1926.67 |  |  | X |  |  |
| 1938.68 |  | X |  |  |  |
| 1954.68 | Hex5HexNAc4NeuAc1 + 1Na | X | X | 1954.6768 | 1.637 |
| 1957.69 |  | X |  |  |  |
| 1960.66 |  | X |  |  |  |
| 1970.65 |  |  | X |  |  |
| 1976.66 | Hex5HexNAc4NeuAc1 + 2Na | X | X | 1976.6588 | 0.607 |
| 1992.63 |  |  | X |  |  |
| 1994.71 |  | X |  |  |  |
| 1996.72 |  | X | X |  |  |
| 2010.70 |  |  | X |  |  |
| 2012.72 | Hex5dHex1HexNAc5 + 1Na | X | X | 2012.7187 | 0.646 |
| 2021.67 |  | X | X |  |  |
| 2028.71 | Hex6HexNAc5 + 1Na | X | X | 2028.7136 | -1.775 |
| 2038.74 |  | X |  |  |  |
| 2044.69 |  |  | X |  |  |
| 2054.73 |  | X | X |  |  |
| 2056.74 |  | X | X |  |  |
| 2067.69 | Hex10HexNAc2 + 1Na | X |  | 2067.6866 | 1.644 |
| 2072.73 |  |  | X |  |  |
| 2082.73 |  | X | X |  |  |
| 2100.74 | Hex5dHex1HexNAc4NeuAc1 + 1Na | X | X | 2100.7347 | 2.523 |
| 2116.71 |  |  | X |  |  |
| 2122.72 | Hex5dHex1HexNAc4NeuAc1 + 2Na | X | X | 2122.7167 | 1.555 |
| 2138.69 |  |  | X |  |  |
| 2141.76 | Hex4dHex1HexNAc5NeuAc1 + 1Na | X |  | 2141.7613 | -0.607 |
| 2158.77 | Hex5dHex2HexNAc5 + 1Na | X | X | 2158.7766 | -3.057 |
| 2163.74 |  |  |  |  |  |
| 2172.76 |  |  | X |  |  |
| 2174.77 | Hex6dHex1HexNAc5 + 1Na | X | X | 2174.7715 | -0.690 |
| 2190.75 |  |  | X |  |  |
| 2215.80 | Hex5dHex1HexNAc6 + 1Na | X | X | 2215.798 | 0.903 |
| 2245.77 | Hex5HexNAc4NeuAc2 + 1Na | X |  | 2245.7722 | -0.980 |
| 2259.82 |  | X |  |  |  |
| 2267.75 |  | X |  |  |  |
| 2275.81 |  | X |  |  |  |
| 2289.74 | Hex5HexNAc4NeuAc2 + 3Na | X |  | 2289.7361 | 1.703 |
| 2303.82 |  | X |  |  |  |
| 2319.81 |  | X | X |  |  |
| 2325.80 | Hex5dHex1HexNAc5NeuAc1 + 2Na | X |  | 2325.7961 | 1.677 |
| 2377.85 | Hex6dHex1HexNAc6 + 1Na | X | X | 2377.8509 | -0.378 |
| 2391.83 |  | X |  |  |  |
| 2393.84 |  |  |  |  |  |
| 2413.81 |  | X |  |  |  |
| 2421.88 |  | X | X |  |  |
| 2435.79 | Hex5dHex1HexNAc4NeuAc2 + 3Na | X |  | 2435.794 | -1.642 |
| 2465.87 |  | X | X |  |  |
| 2487.85 | Hex6dHex1HexNAc5NeuAc1 + 2Na | X |  | 2487.8489 | 0.442 |
| 2539.91 | Hex7dHex1HexNAc6 + 1Na | X |  | 2539.9037 | 2.480 |


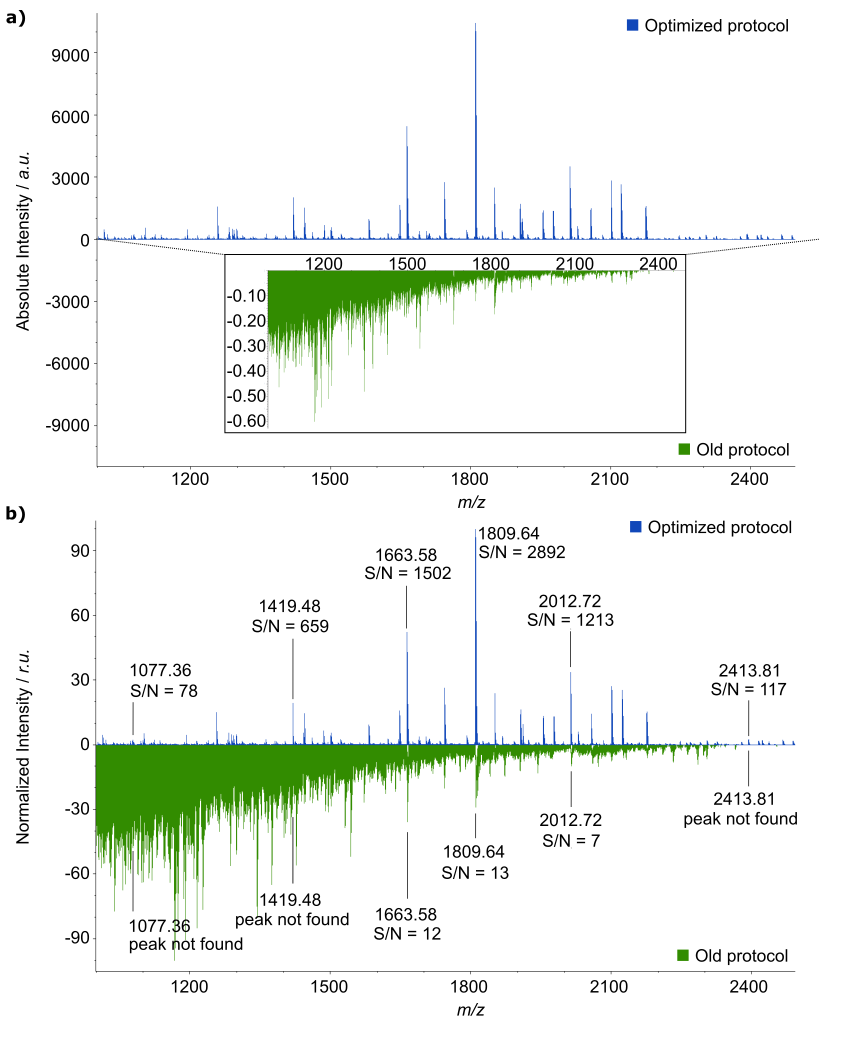


# **Figure S1**: Comparison of MALDI MSI spectra from N-glycan imaging comparing the old protocol with the new optimized protocol for fresh frozen imaging. (**a**) Absolute intensity spectra. (**b**) Normalized intensity spectra.

**
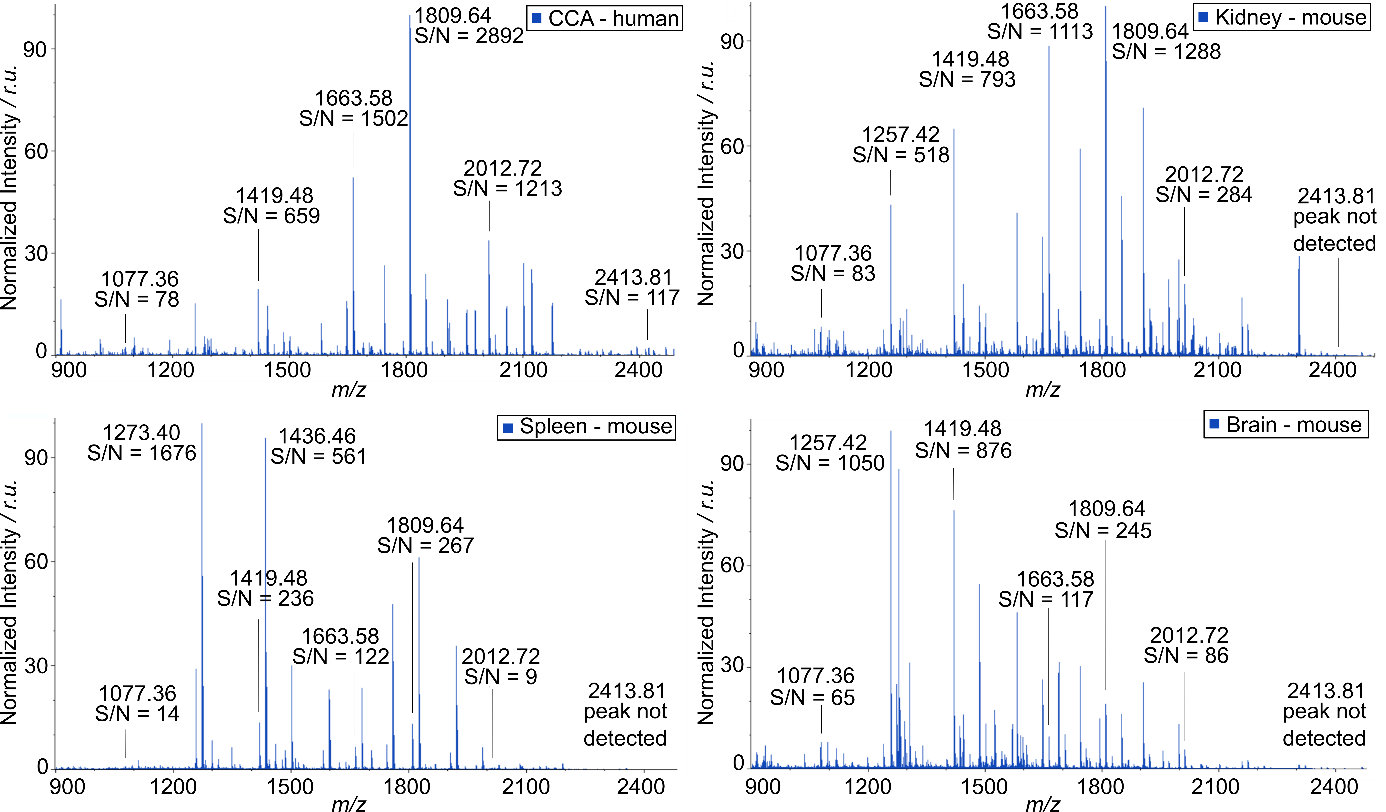
**

**Figure S2**: MALDI MSI spectra after optimized on-tissue N-glycan digestion protocol on four different fresh frozen tissue types. Mass spectra has been normalized to the total ion count.

**
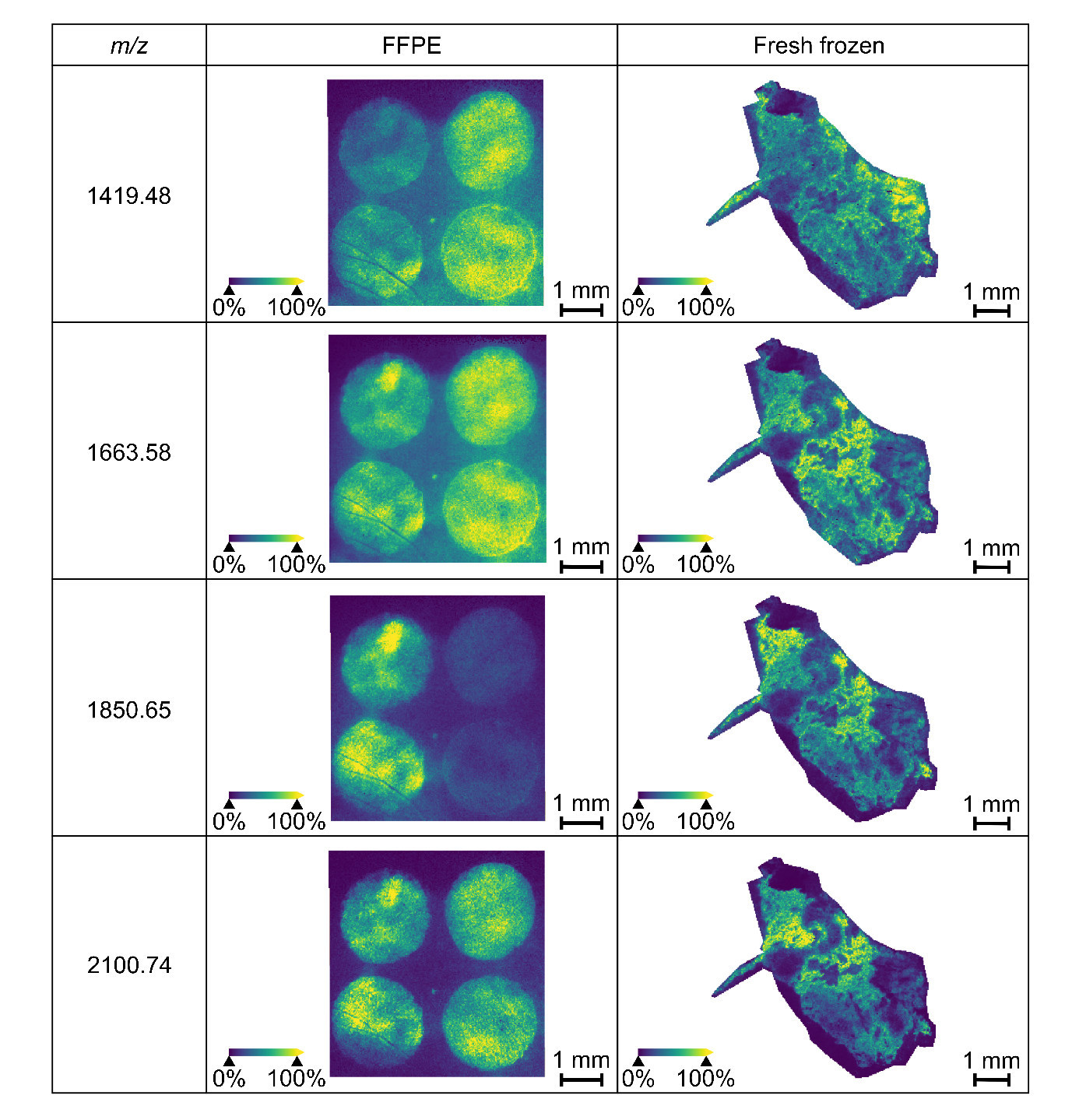
**

# **Figure S3:** Comparison of delocalization between MALDI MSI measurements of N-glycans on fresh frozen and FFPE tissue sections.


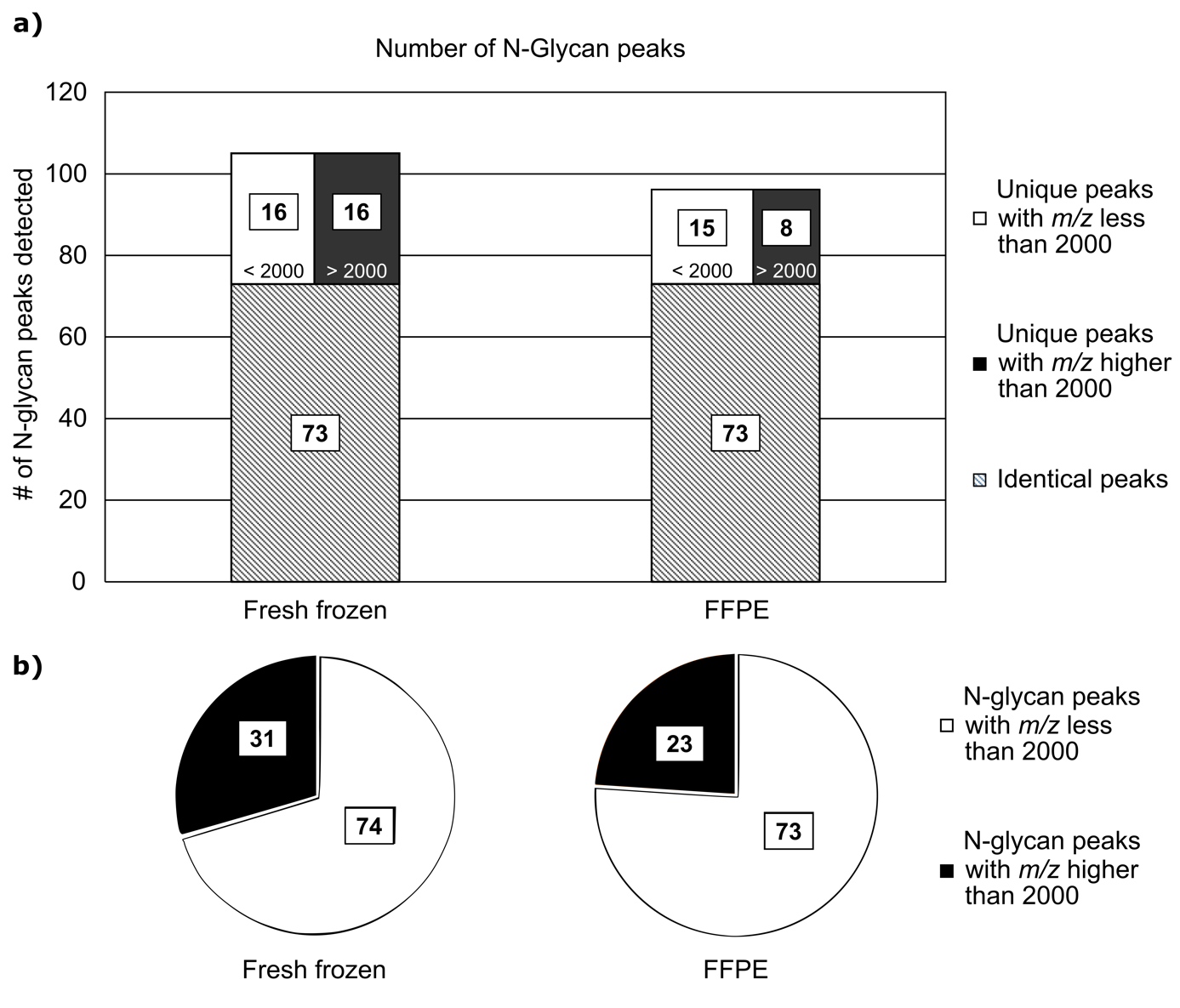


# **Figure S4**: Overview of potential N-glycans detected across the m/z-range. (a) Bar graph of the number of potential N-Glycan peaks detected in fresh frozen and FFPE tissue. (b) Pie chart of the number of potential N-Glycan peaks detected with m/z-value less or higher than 2000 in fresh frozen and FFPE tissue.


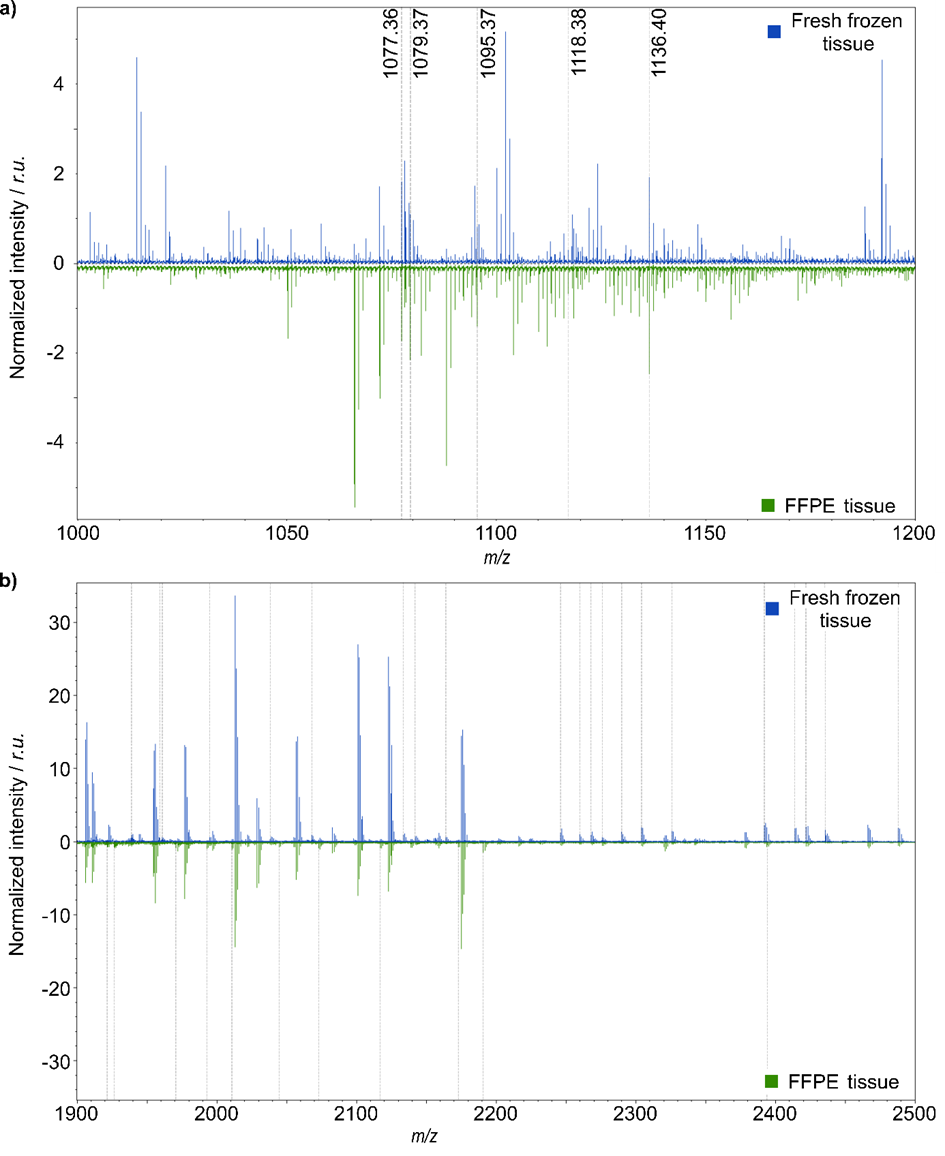


# **Figure S5**: Low (a) and high (b) m/z-range of normalized average mass spectra acquired from fresh frozen CCA tissue sections (top, blue) and FFPE tissue TMA of PDAC/CCA (bottom, green). In figure (a) all N-glycan peaks in both mass spectra are highlighted with a dashed grey vertical line. In figure (b) unique N-glycan peaks in both mass spectra are highlighted with a grey dashed vertical line.
